# Medical and End-of-Life Decision-Making Preferences in Adolescents and Young Adults with Advanced Heart Disease and Their Parents

**DOI:** 10.1001/jamanetworkopen.2023.11957

**Published:** 2023-05-05

**Authors:** Melissa K. Cousino, Victoria A. Miller, Cynthia Smith, Heang M. Lim, Sunkyung Yu, Ray Lowery, Karen Uzark, Emily M. Fredericks, Joanne Wolfe, Elizabeth D. Blume, Kurt R. Schumacher

**Affiliations:** 1 Department of Pediatrics, Michigan Medicine, Ann Arbor; 2 Department of Cardiac Surgery, Michigan Medicine, Ann Arbor; 3 Division of Adolescent Medicine, Children’s Hospital of Philadelphia, Philadelphia, Pennsylvania; 4 Department of Pediatrics, Boston Children’s Hospital, Boston, Massachusetts

## Abstract

**Importance:**

Despite high disease morbidity and mortality and complex treatment decisions, little is known about the medical and end-of-life decision-making preferences of adolescents and young adults (AYA) with advanced heart disease. AYA decision-making involvement is associated with important outcomes in other chronic illness groups.

**Objective:**

To characterize decision-making preferences of AYAs with advanced heart disease and their parents and determine factors associated with these preferences.

**Design, Setting, and Participants:**

Cross-sectional survey between July 2018 and April 2021 at a single-center heart failure/transplant service at a Midwestern US children’s hospital. Participants were AYAs aged 12 to 24 years with heart failure, listed for heart transplantation, or posttransplant with life-limiting complications and a parent/caregiver. Data were analyzed from May 2021 to June 2022.

**Main Outcomes and Measures:**

Single-item measure of medical decision-making preferences, MyCHATT, and Lyon Family-Centered Advance Care Planning Survey.

**Results:**

Fifty-six of 63 eligible patients enrolled in the study (88.9%) with 53 AYA-parent dyads. Median (IQR) patient age was 17.8 (15.8-19.0) years; 34 (64.2%) patients were male, and 40 patients (75.5%) identified as White and 13 patients (24.5%) identified as members of a racial or ethnic minority group or multiracial. The greatest proportion of AYA participants (24 of 53 participants [45.3%]) indicated a preference for active, patient-led decision-making specific to heart disease management, while the greatest proportion of parents (18 of 51 participants [35.3%]) preferred they and physician(s) make shared medical decisions on behalf of their AYA, representing AYA-parent decision-making discordance (χ^2^ = 11.7; *P* = .01). Most AYA participants stated a preference to discuss adverse effects or risks of treatment (46 of 53 participants [86.8%]), procedural and/or surgical details (45 of 53 participants [84.9%]), impact of condition on daily activities (48 of 53 participants [90.6%]), and their prognosis (42 of 53 participants [79.2%]). More than half of AYAs preferred to be involved in end-of-life decisions if very ill (30 of 53 participants [56.6%]). Longer time since cardiac diagnosis (*r* = 0.32; *P* = .02) and worse functional status (mean [SD] 4.3 [1.4] in New York Heart Association class III or IV vs 2.8 [1.8] in New York Heart Association class I or II; t-value = 2.7; *P* = .01) were associated with a preference for more active, patient-led decision-making.

**Conclusions and Relevance:**

In this survey study, most AYAs with advanced heart disease favored active roles in medical decision-making. Interventions and educational efforts targeting clinicians, AYAs with heart disease, and their caregivers are needed to ensure they are meeting the decision-making and communication preferences of this patient population with complex disease and treatment courses.

## Key Points

**Question:**

What are the medical and end-of-life decision-making preferences of adolescents and young adults (AYA) with advanced heart disease?

**Findings:**

In this cross-sectional survey study of 56 patients, the majority of AYA participants stated a preference to discuss adverse effects or risks of treatment (87%), procedural/surgical details (85%), prognosis (79%), and end-of-life care wishes (57%). AYAs preferred more patient-led active decision-making while parents preferred more parent/physician shared decision-making, suggesting significant AYA-parent discordance.

**Meaning:**

These findings suggest interventions to better meet the decision-making needs of AYAs with advanced heart disease are warranted.

## Teaser

This cross-sectional study evaluates the medical decision-making preferences of adolescents and young adults with advanced heart disease and their parents.

## Introduction

Over the past decade, the field of pediatric heart failure has experienced tremendous change, from a growing patient population^1^ to increasing use of advanced cardiac therapies, such as ventricular assist devices.^2^ Despite advancements in treatment, morbidity and mortality in pediatric heart failure remain high.^1,3^ Children with heart failure are more likely to die in the emergency department or during a hospitalization (4.3%) when compared with hospitalized peers without heart failure (0.04%) and adults with heart failure.^1,3^ A majority of parents of children with advanced heart disease report considerable symptom burden in their child,^4^ and one-third feel underprepared to make medical decisions regarding their child’s medical care.^5^

Many have sought to understand and describe the health care communication and medical decision-making preferences of young people with serious, life-threatening illnesses given the potential for invasive interventions and/or shortened life expectancy. A collective literature review suggests many adolescents and young adults (AYAs) with oncologic diseases desire prognostic information early in their disease course, want to be made aware of their treatment options, and desire shared decision-making with caregivers.^6,7,8^ It has also been reported that a majority of AYAs with cancer describe a preference to discuss their end-of-life care wishes—and to do so before becoming acutely unwell.^9^ Despite high disease burden, intensive treatment demands, and notable risk for death, the health care communication and medical decision-making preferences of youth with advanced heart disease and heart failure are largely unknown. In a pilot sample of 12 adolescents listed for heart transplantation, the majority of respondents expressed a desire to discuss their prognosis (83%) and end-of-life wishes (67%), as well as engage in their end-of-life decision-making if critically ill (83%).^10^ Yet, surveys of pediatric cardiology clinicians,^11^ patients, and parents^10^ suggest that these health care communication and medical decision preferences are often unaddressed in AYAs with advanced heart disease.

Research on patient communication and medical decision-making was recently identified as a top priority for investigation specific to palliative and end-of-life care in pediatric cardiology,^12^ consistent with a National Academy of Medicine call for research on communication and shared decision-making in AYA serious illness.^13^ The current study aimed to characterize communication and medical decision-making preferences of AYAs with advanced heart disease and determine factors associated with these preferences to inform the timing, targets, and potential outcomes of future interventions. Patient preferences were compared with parent/caregiver decision-making preferences, as well as parent/caregiver perceptions of what their child desired in terms of communication and decision-making engagement. It was hypothesized that AYAs would desire greater involvement in medical decision-making than their parents preferred.

## Methods

### Study Design and Population

This federally funded, single-center, cross-sectional study was approved by the University of Michigan Medical School institutional review board. All participants provided written assent or consent. This study followed the Strengthening the Reporting of Observational Studies in Epidemiology (STROBE) reporting guideline. Eligible participants included patients aged 12 to 24 years and a parent/guardian. Medical inclusion criteria for advanced heart disease was informed by other studies of this population,^5,14^ but purposefully broader to capture patients across the disease continuum. Those with any of the following were eligible: (1) American Heart Association stage c heart failure or higher, (2) receiving chronic medical therapy for heart failure, (3) listed for heart transplantation, or (4) post–heart transplantation with life-limiting complication (eg, cardiac allograft vasculopathy, refractory rejection). Exclusion criteria included (1) suicidality, homicidality, or psychosis in past 6 months, (2) intubated, unable to respond verbally, or with active delirium, (3) intellectual impairment or significant developmental delay, and (4) non-English speaking due to lack of translated study measures. A subanalysis of quality of life in this sample was described in an earlier manuscript.^15^

Participants were identified by study coordinator via weekly review of outpatient heart failure/heart transplant clinic schedule and inpatient heart failure/heart transplant patient list. Eligibility was verified by study principal investigator or co-investigator. Participants were recruited using multiple methods, including face-to-face recruitment during outpatient clinic visits and inpatient medical stays, as well as via telephone. Participant recruitment occurred July 2018 to April 2021, with an extended period of pause in recruitment as a result of the COVID-19 pandemic.

### Measures

Participating dyads completed all measures electronically. An ordinally scaled single-item measure was used to assess preferences for adolescent involvement in medical decision-making.^16^ Item selections were further categorized as passive (ie, defer to physician or parent), shared (ie, together with physician or parent), or active (ie, patient-led decision-making) decision-making preference. The MyCHATT tool^17^ was also used to describe AYA communication and decision-making preferences regarding specific topics (ie, discuss adverse effects, discuss procedural/surgical details, discuss prognosis), while 12 nominally scaled questions from the Lyon Family-Centered Advance Care Planning Survey^18,19^ were used to assess AYA participants’ preferences specific to involvement in advance care planning and end-of-life decision-making. Accompanying parent versions of these measures were used to gauge parental preferences on overall decision-making (single-item scale) as well as parental perceptions on their child’s communication and decision-making preferences regarding specific topics (ie, MyCHATT and Lyon Family-Centered Advance Care Planning Survey). Self-reported sociodemographic characteristics were collected from adult participants (patients aged ≥18 years or parent/caregivers). Race and ethnicity were assessed to determine generalizability of results. Patient sex, age at survey, cardiac diagnosis, age at diagnosis, cardiac device (eg, ventricular assist device, implantable cardiac device) and surgical history, hospitalizations, New York Heart Association (NYHA) functional class, and palliative care involvement were obtained via medical record review.

### Statistical Analysis

Before starting study recruitment, a sample size calculation determined 46 AYA-parent dyads were needed to detect a medium effect size (correlation coefficient, *r* = 0.40) of the hypothesized associations to achieve an 80% power with a 2-sided 0.05 significance level.^15^ Standard descriptive statistics were reported as frequency with percentage for categorical variables and median (IQR) or mean (SD), depending on distributional assumption, for continuous variables. Univariate associations of patient demographic and medical history with AYA decision-making preferences by an AYA-reported single-item measure with an ordinal scale were evaluated to explore factors associated with AYA decision-making preferences, using 2-sample *t* test or analysis of variance for categorical variables and Spearman correlation coefficient (*r*) for continuous variables. The overall difference in decision-making preferences (passive, shared, or active) between AYA participants and their parents was examined using McNemar-Bowker test and was also presented by AYA participants’ age at survey (<18 years vs ≥18 years). All analyses were performed using SAS version 9.4 (SAS Institute), with a statistical significance level of .05 using 2-sided tests. Data were analyzed from May 2021 to June 2022.

## Results

### Participant Characteristics

The final study sample included 53 AYA-parent dyads. Median (IQR) patient age was 17.8 (15.8-19.0) years and 34 patients (64.2%) were male. The majority of patient participants identified as White (40 patients [75.5%]); 13 patients (24.5%) identified as members of a minority racial group, biracial, or multiracial. Primary cardiac diagnosis and disease characteristics are detailed in Table 1. Parent respondents were primarily female (43 respondents [84.3%]). Participant characteristics are reported in Table 1 and detailed in an earlier report from this data set.^15^

**Table 1.** Adolescent and Young Adult Patient Sociodemographic and Disease Characteristics

Characteristic	Patients, No. (%) (N = 53)
Sex	
Male	34 (64.2)
Female	19 (35.8)
Patient age at survey, median (IQR), y	17.8 (15.8-19.0)
Patient race and ethnicity	
Biracial/multiracial	3 (5.7)
Black/African American	7 (13.2)
Hispanic or Latinx	2 (3.8)
American Indian	1 (1.9)
White	40 (75.5)
Patient’s cardiac diagnosis	
Dilated cardiomyopathy	9 (17.0)
Restrictive cardiomyopathy	0 (0.0)
Hypertrophic cardiomyopathy	2 (3.8)
Anthracycline cardiomyopathy	11 (20.8)
Other cardiomyopathy	2 (3.8)
CHD—single ventricle	12 (22.6)
CHD—not single ventricle	3 (5.7)
Posttransplant with complications	14 (26.4)
Age at cardiac diagnosis, median (IQR), y	7.7 (0.0-14.3)
ECMO history	5 (9.4)
Cardiac device history	12 (22.6)
Cardiac arrest history	4 (7.5)
Extracardiac disease	22 (41.5)
Cardiac surgery (not including heart transplant)	21 (39.6)
Catheterization interventions within the last 5 y	18 (34.0)
Hospitalizations within the last 5 y	40 (75.5)
New York Heart Association class	
I or II	20 (37.7)
III or IV	15 (28.3)
Unknown	18 (34.0)
Heart transplant recipient	14 (26.4)
Resuscitation	53 (100.0)
Referral to palliative care team	8 (15.1)
Family type	
Single-parent home	12 (22.6)
Married, both parents live at home	31 (58.5)
Mixed family home	9 (17.0)
Other	1 (1.9)
Family’s annual income, $	
<25 000	6 (11.3)
25 000-49 999	13 (24.5)
50 000-74 999	2 (3.8)
75 000-100 000	10 (18.9)
>100 000	21 (39.6)
Not reported	1 (1.9)
Highest level of education completed by the patient’s mother	
Some high school	2 (3.8)
High school	7 (13.2)
Some college	24 (45.3)
Bachelor’s degree	12 (22.6)
Professional degree (master’s, doctorate degree)	8 (15.1)
Highest level of education completed by the patient’s father	
Some high school	4 (7.5)
High school	14 (26.4)
Some college	16 (30.2)
Bachelor’s degree	14 (26.4)
Professional degree (master’s, doctorate degree)	5 (9.4)

Abbreviations: CHD, congenital heart disease; ECMO, extracorporeal membrane oxygenation.

During the recruitment period, 100 participants were identified as meeting medical eligibility. Among these, 37 were deemed ineligible due to cognitive/neurodevelopmental status of patient prohibiting survey completion (26 patients), non-English speaking patient or caregiver (3 patients), suicidal ideation within recent 6 months (3 patients), heart transplant or death before enrollment (3 patients), or cardiologist did not support approaching due to patient/family emotional or health state (2 patients). Fifty-six of the remaining 63 eligible patients enrolled in the study (88.9% enrollment rate). Three consenting participants were lost to follow-up for a total sample of 53 AYA-parent dyads reported here.

### AYA-Reported Preferences

**Figure 1.**
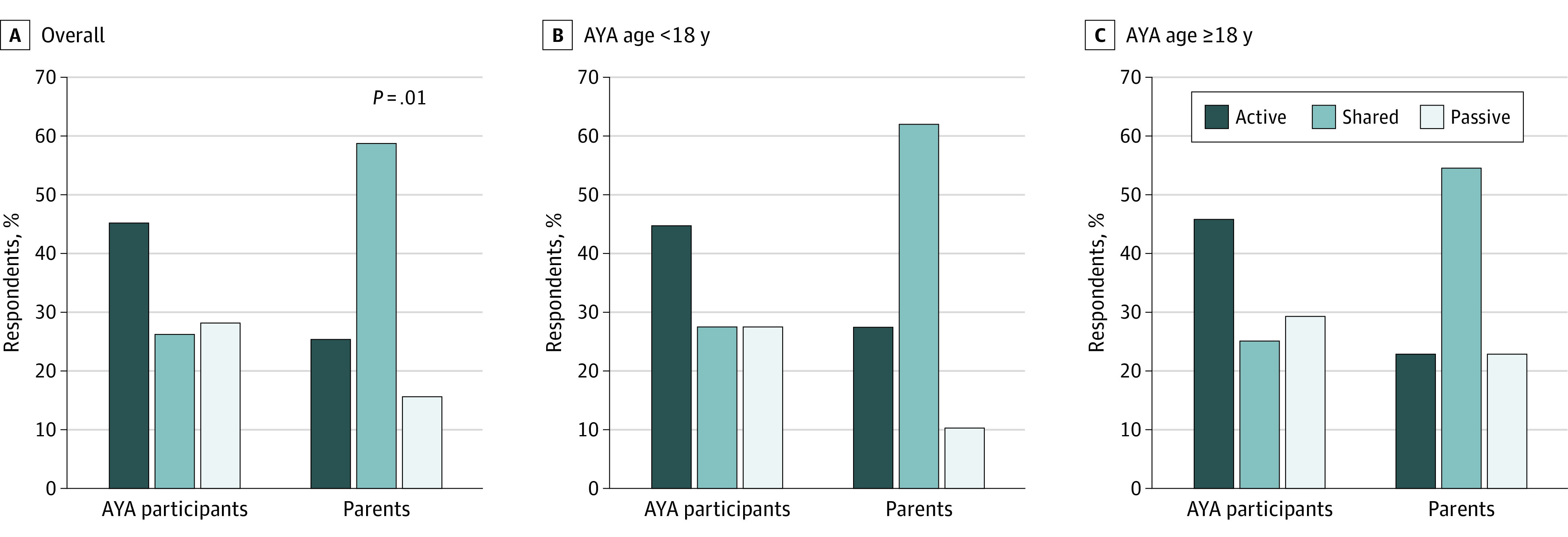
Adolescent and Young Adult (AYA) and Parent Medical Decision-Making Preferences The graphs show the preferences of AYAs and their parents as to whether AYAs should have an active role, a passive role, or share decision-making with their parents and/or physicians overall and by age group.

Overall, the greatest proportion of AYAs (24 of 53 participants [45.3%]), indicated a preference for active, patient-led decision-making specific to heart disease management (Figure 1). Specifically, 41.5% of AYA participants (22 of 53 participants) selected the following: “I should make the decision but strongly consider the physician(s)’ and my parents(s)’ opinions.” Passive decision-making was preferred by 28.3% of AYA participants (15 of 53 participants), with the smallest proportion (10 of 53 participants [18.9%]) preferring their physician(s) make the medical decision as guided by physician knowledge of the disease and treatment. Zero AYA participants selected the following response, “My parent(s) should make the decision using all that he/she knows about the treatment,” and 3 participants (5.7%) indicated a preference for parent decision-making with strong consideration of AYA patient opinion. Approximately a quarter of the AYA participants (xx patients [26.5%]) indicated a preference for shared decision-making with parent(s) or physician(s).

**Figure 2.**
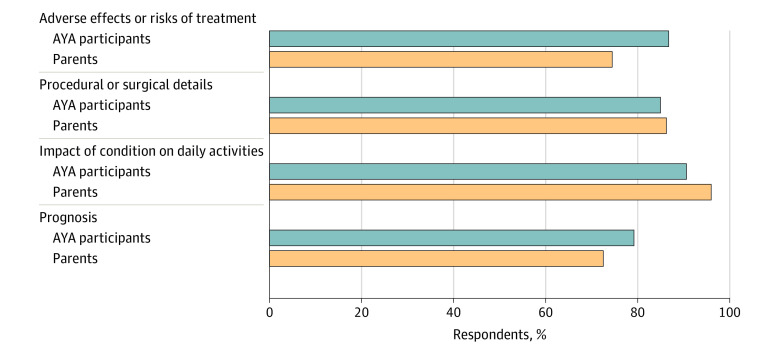
AYA Communication Preferences and Parent-Perceived AYA Communication Preferences The graph shows the percentage of adolescent and young adult (AYA) participants and their parents who believed that AYAs should be involved in discussing each aspect of the AYA's illness and treatment.

**Figure 3.**
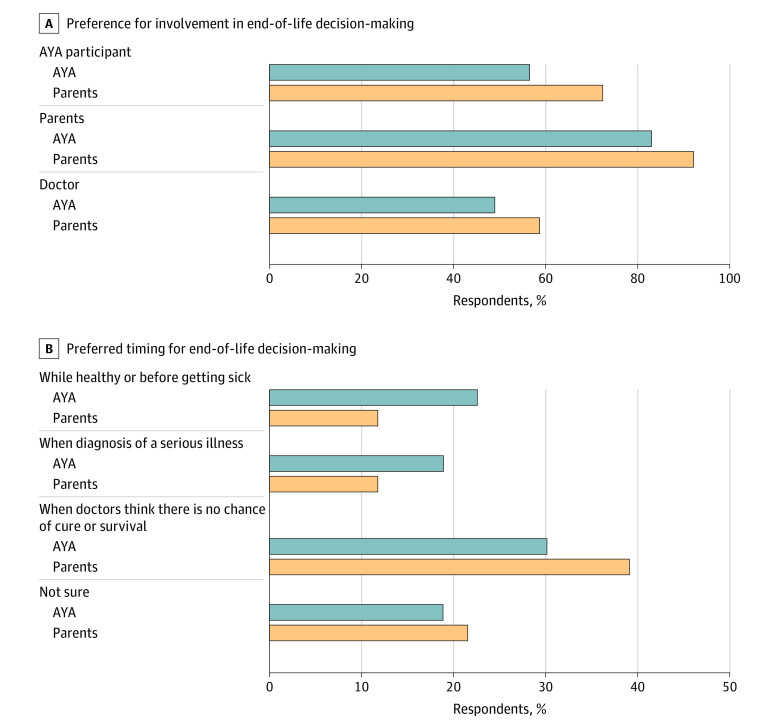
Preferences for End-of-Life Decision-Making The graphs show adolescent and young adult (AYA) patient and parent preferences for AYA involvement in end-of-life discussions (A) and the preferred timing of these discussions (B).

Overall, the majority of AYA participants stated a preference to discuss adverse effects or risks of treatment (46 of 53 participants [86.8%]), procedural/surgical details (45 of 53 participants [84.9%]), impact of condition on daily activities (48 of 53 participants [90.6%]), and their prognosis (42 of 53 participants [79%]) (eFigure 1 in Supplement 1 and Figure 2). With regards to end-of-life decision-making involvement, 30 of 53 AYAs (56.6%) stated a preference to be involved in these decisions if very ill. The majority of participants also desired parental involvement in their end-of-life decision-making (44 of 53 participants [83.0%]) while approximately half (26 of 53 participants [49.1%]) indicated a preference for physician involvement (eFigure 2 in Supplement 1 and Figure 3A). Preferred timing for end-of-life decision-making discussions was variable across AYA participants with 12 of 53 participants (22.6%) stating while healthy/before becoming ill, 10 of 53 participants (18.9%) stating upon diagnosis of a serious illness, and 16 of 53 participants (30.2%) stating when physicians think there is no chance of cure or survival (eFigure 3 in Supplement 1 and Figure 3B). Notably, 10 of 53 participants (18.9%) were unsure of preferred timing for end-of-life decision-making discussions and 14 of 53 participants (26.4%) did not know the typical course or prognosis for their advanced heart disease. The majority of AYA participants felt their parent(s) should initiate conversations about end-of-life decision-making (46 of 53 participants [86.8%]), followed by their cardiologist initiating discussions (27 of 53 participants [50.9%]).

### Associations of Demographic and Medical History with AYA Decision-Making Preferences

AYA sex, age at study, race, and ethnicity were unrelated to decision-making preference (Table 2). Greater time since cardiac diagnosis (*r* = 0.32; *P* = .02) and worse functional status (mean [SD] 4.33 [1.35] in NYHA class III or IV vs 2.8 [1.82] in NYHA class I or II; t-value = 2.74; *P* = .01) were associated with a preference for more active AYA involvement in decision-making. There was no statistically significant difference in preference for an active role in decision-making among patients with congenital heart disease compared with those with cardiomyopathies or posttransplant complications (mean [SD] 4.27 [1.22] vs 3.29 [1.76]; t-value = 1.96; *P* = .06). Other notable medical experiences, such as extracorporeal membrane oxygenation history, cardiac arrest history, cardiac device history, surgical history, and number of hospitalizations within the last 5 years were unrelated to decision-making preferences (Table 2).

**Table 2.** Associations of Patient Demographics and Medical History with Patient Decision-Making Preferences (Using the Single-Item Ordinal 6-Point Scale)^a^

Characteristics	Patient decision-making preference, mean (SD)	*P* value^b^
Sex		
Male	3.68 (1.77)	.53
Female	3.37 (1.54)	
Age at survey, y (*r* = 0.005)		.97
<18	3.59 (1.70)	.92
≥18	3.54 (1.70)	
Race and ethnicity		
White	3.80 (1.60)	.08
Biracial/multiracial	2.85 (1.77)	
Black/African American		
Hispanic/Latinx		
American Indian		
Patient’s cardiac diagnosis		.28
Dilated, hypertrophic or other cardiomyopathy	3.31 (1.75)	.06^c^
Anthracycline cardiomyopathy	3.09 (2.07)	
CHD	4.27 (1.22)	
Posttransplant with complications	3.43 (1.65)	
Age at cardiac diagnosis, y (*r* = −0.28)	NA	.04
Time since cardiac diagnosis, y (*r* = 0.32)	NA	.02
ECMO history		
Yes	4.40 (0.89)	.25
No	3.48 (1.73)	
Cardiac device history		
Yes	3.42 (1.56)	.73
No	3.61 (1.73)	
Cardiac arrest history		
Yes	3.00 (0.82)	0.49
No	3.61 (1.73)	
Extracardiac disease		
Yes	3.32 (1.70)	.37
No	3.74 (1.67	
Cardiac surgery (not including heart transplant)		
Yes	4.00 (1.48)	.13
No	3.28 (1.76)	
Catheterization interventions within the last 5 y		
Yes	3.72 (1.64)	.63
No	3.49 (1.72)	
Hospitalizations within the last 5 y		
Yes	3.40 (1.68)	.21
No	4.08 (1.66)	
New York Heart Association class (n = 35)		
I or II	2.80 (1.82)	.01
III or IV	4.33 (1.35)	
Heart transplant recipient		
Yes	3.43 (1.65)	.73
No	3.62 (1.71)	
Referral to palliative care team		
Yes	2.88 (2.03)	.21
No	3.69 (1.61)	

Abbreviations: CHD, congenital heart disease; ECMO, extracorporeal membrane oxygenation; NA, not applicable.

^a^ Single-item ordinal scale used to measure patient decision-making; higher score indicates preference for more active decision-making involvement.

^b^ *P* value from 2 sample *t* test or analysis of variance for categorical variables and Spearman correlation coefficient for continuous variables.

^c^ Comparison was made as CHD (mean [SD] 4.27 [1.22]) vs all others (mean [SD] 3.29 [1.76]) and *P* value came from 2-sample *t* test.

### Parent-Reported Preferences

Notably, about one-third of parents (18 of 51 participants [35.3%]) felt they and their child’s physician(s) should make shared medical decisions on behalf of their AYA. A quarter of parents (12 of 51 participants [23.5%]) reported they and their AYA should make medical decisions together, while 6 of 51 parents (11.8%) believed their AYA should make their own medical decisions.

### Parent Perceptions of AYA Preferences

Most parents perceived their AYA would want to discuss adverse effects or risks of treatment (38 of 51 participants [74.5%]), procedural/surgical details (44 of 51 participants [86.3%]), impact of their condition on daily activities (49 of 51 participants [96.1%]), and their prognosis (37 of 51 participants [72.5%]) (Figure 2). Overall, 37 of 51 parents (72.5%) perceived that their AYA would desire involvement in their own end-of-life decision-making, as well as involvement from parent(s) (47 of 51 participants [92.2%]) and their physician(s) (30 of 51 participants [58.8%]) in this type of decision-making (Figure 3A). The highest percentage of parents believed their AYA would want to engage in end-of-life decision-making when there is no chance of cure or survival (20 of 51 participants [39.2%]) (Figure 3B). Similar to patients, most parents felt that they (40 of 51 participants [78.4%]) and and/or their child’s cardiologist (32 of 51 participants [62.7%]) should initiate end-of-life decision-making discussions.

### AYA and Parent Agreement in Decision-Making Preferences

Consistent with hypotheses, there was a significant difference in overall decision-making preferences between AYA and parent preferences (χ^2^ = 11.7; *P* = .01, Figure 1A). Overall, AYAs were more likely to desire active, patient-led decision-making, while their parents were most likely to desire shared decision-making. This was similar by AYA age groups (<18 years [Figure 1B] and ≥18 years [Figure 1C]).

## Discussion

Highlighted as one of the highest priorities for research within pediatric cardiology palliative care-focused science,^12^ this is the first study we know of to describe the communication and decision-making preferences of the growing population of AYAs with advanced heart disease. We found that most AYAs with advanced heart disease favor active patient-led roles in medical decision-making while the greatest proportion of parents have a preference for shared medical decision-making between parent/physician. Moreover, most AYAs desire communication about adverse effects or risks of treatment, procedural/surgical details, impact of condition on daily activities, and their prognosis. More than half of AYAs stated a preference to be involved in end-of-life decisions if very ill.

The vast majority of AYAs with advanced heart disease desire a high level of active engagement in their medical decision-making and prefer to be informed about risks, adverse effects, impact of condition on daily activities, and their prognosis. This is consistent with research conducted among AYAs with cancer.^6,9^ In a study of more than 150 AYAs aged 11 to 19 years with a chronic illness, the majority of respondents preferred direct communication from their physician regarding their health care.^20^ In the current study, AYAs with greater time since cardiac diagnosis and those experiencing more notable symptom burden were most likely to desire active involvement in their medical decision-making. Other work has suggested that those with more advanced illness may desire more involvement in medical decision-making. For example, in a sample of AYAs with noncurative cancer, 85% reported that they made the final decision to enroll in a phase 1 clinical trial.^21^ It may be the case that opportunities to participate in medical decision-making are more regularly offered by clinicians and parents of children and AYAs with longer illness experience or worsened disease status.

More than half of AYAs also stated a desire to be involved in end-of-life decision-making if very ill, which is consistent with a pilot sample of AYAs actively listed for heart transplantation, in which 83% stated a preference to be involved in their end-of-life decision-making.^10^ Despite these patient preferences, prior research suggests the field is not regularly meeting these needs.^11^ Decision-making support and goals of care discussions are foundational to palliative care. When compared with other serious illness groups, palliative care teams are not as regularly involved in the care of children with cardiac diseases.^22^ In a single-center retrospective analysis of children who died of advanced heart disease, only 16% received a subspeciality palliative care consultation. Although three-quarters of the medical records of patients in this study indicated an end-of-life discussion was had with parents,^23^ other studies have highlighted the risk of sudden, unexpected death in some advanced heart disease populations,^24,25^ leaving little time for such discussions with patients themselves. Palliative care–focused discussions, whether by cardiac team or subspeciality palliative care team, are likely to result in communication and decision-making approaches best aligned with patient preferences. This has been evidenced by a single-center experience demonstrating that earlier palliative care involvement for pediatric patients with VAD resulted in more frequent discussions about goals and of care and end-of-life care planning.^26^

Notably, the current study also demonstrated considerable discordance between AYA decision-making preferences and parent preferences specific to their child’s medical decision-making. Although it is regularly assumed that parent and family caregivers have a good sense of AYA preferences and goals specific to medical and end-of-life decision-making,^8^ empirical research has demonstrated both a lack of congruence and awareness in AYA-parent decision-making preferences.^9,27,28^ For example, across a multicenter study of AYAs with cancer, 89% of AYAs preferred to discuss end-of-life decision-making early in their disease course yet only 39% of families were aware of such preferences.^27^ Certainly, many well-intentioned parents are understandably attempting to “protect” their children from difficult information, conversations, and decision-making, which may contribute to the discordance observed in this study and others. It is also possible that communication gaps between clinicians and parents^14^ contribute to AYA-parent decision-making discordance. For example, if parents/caregivers lack complete understanding of their child’s disease course and treatment options themselves, it may be more challenging to involve their AYA in medical decisions.

Taken together, study findings have important implications for clinicians caring for children and AYAs with serious forms of heart disease. First, it is imperative that communication gaps be addressed. Both clinicians and parents/caregivers may be underestimating the degree to which AYAs want to be actively involved in their heart disease decision-making. The current study underscores the importance of assessing communication and decision-making preferences early in the cardiac disease course, which is in line with the recent American Heart Association clinical practice guideline on palliative care in pediatric cardiology.^29^ Consistent with other studies,^28^ few demographic and medical variables were associated with AYA decision-making preferences in the current study. As such, individualized assessment of communication and decision-making preferences should occur for each unique patient encounter, regardless of diagnosis, disease state, age, or symptom burden. Conversation tools have been developed to assess youth communication and decision-making preferences,^17^ as well as clinician-^30^ and family-directed^30,31^ pediatric and AYA advance care planning interventions. As the cardiac disease course is often different from other pediatric illness groups where such interventions have been more regularly tested, it will be important to revise, develop, and pilot communication and decision-making interventions with the advanced heart disease patient population.

Second, bolstering training and educational programs for cardiology clinicians specific to AYA communication and decision-making in heart disease care and at end-of-life is an important next step.^29^ In a survey of pediatric cardiologists, only one-third endorsed competence prognosticating life expectancy and two-thirds indicated competence in caring for children with heart disease at end of life.^32^ With a majority of AYAs in the current study stating a preference to discuss their prognosis and end-of-life decision-making, better preparing cardiology clinicians to have these conversations, even amidst uncertainty, will be helpful in meeting AYA preferences.

### Limitations

There are important limitations to consider when interpreting the study’s results. First, this is a single-center study. Despite excellent enrollment rates and diverse representation across cardiac diagnoses and patient race, heart center practices and culture may influence patient and parent communication and decision-making preferences. For example, some centers standardly consult palliative care upon extracorporeal membrane oxygenation initiation or heart transplant referral,^32,33^ which may prompt additional discussions about decision-making preferences and needs. As such, a multicenter investigation of AYA decision-making preferences is needed to better generalize findings reported in the current study. Moreover, although a meaningful subset of participants identified as living at or below the poverty level and/or endorsed limited formal education, additional social determinants of health, including health care access and health literacy, should be measured in future studies to better understand the impact of these critically important factors on communication and decision-making. Some of these data were collected amidst the COVID-19 pandemic. Certainly, changes in health care practices (ie, cancelled appointments, telemedicine), pandemic stressors, and the unknown effects of COVID-19 infection on young people with heart disease^34^ may have impacted survey results. Although a priori power analysis supported the sample size for detecting associations between variables of interest, the single-center sample size limited analyses within groups (eg, by diagnosis, by race) and consideration for adjustment of multiple comparisons. Lastly, communication and decision-making preferences are not always best captured through quantitative, survey-based data. Future qualitative and mixed-methods study will be important for better understanding the intricacies of health care communication with AYAs as well as informing intervention design.

## Conclusions

In this survey study, the majority of AYA participants stated a preference to discuss adverse effects or risks of treatment, procedural/surgical details, prognosis, and end-of-life care wishes. AYAs preferred more patient-led active decision-making while parents preferred more parent/physician shared decision-making, suggesting significant AYA-parent discordance. Clinical interventions and educational efforts targeting clinicians, AYAs with heart disease and their parents/caregivers are needed to ensure we are meeting the decision-making and communication needs of this growing patient population with complex, high-risk heart disease.

## Notes

Supplement 1. **eFigure 1.** AYA Communication Preferences and Parent-Perceived AYA Communication Preferences by AYA Age at Survey **eFigure 2.** Preferences for Involvement in End-of-Life Decision-Making by AYA Age at Survey **eFigure 3.** Preferred Timing for End-of-Life Decision-Making Discussions by AYA Age at Survey

Supplement 2.
Data Sharing Statement
