# Supplementary material for: Medical and End-of-Life Decision-Making Preferences in Adolescents and Young Adults with Advanced Heart Disease and Their Parents
Source: JAMA Netw Open. 2023 May 5;6(5):e2311957. doi: 10.1001/jamanetworkopen.2023.11957 (PMC10163392; doi:10.1001/jamanetworkopen.2023.11957)
Supplement: Supplement 1. — eFigure 1. AYA Communication Preferences and Parent-Perceived AYA Communication Preferences by AYA Age at Survey eFigure 2. Preferences for Involvement in End-of-Life Decision-Making by AYA Age at Survey eFigure 3. Preferred Timing for End-of-Life Decision-Making Discussions by AYA Age at Survey [file jamanetwopen-e2311957-s001.pdf]

Supplemental Online Content

Cousino MK, Miller VA, Smith C, et al. Medical and end-of-life decision-making preferences in adolescents and young adults with advanced heart disease and their parents. *JAMA Netw Open*. 2023;6(5):e2311957. doi:10.1001/jamanetworkopen.2023.11957

eFigure 1. AYA Communication Preferences and Parent-Perceived AYA Communication Preferences by AYA Age at Survey

eFigure 2. Preferences for Involvement in End-of-Life Decision-Making by AYA Age at Survey

eFigure 3. Preferred Timing for End-of-Life Decision-Making Discussions by AYA Age at Survey

This supplemental material has been provided by the authors to give readers additional information about their work.

© 2023 Cousino MK et al. *JAMA Network Open*.

eFigure 1. AYA Communication Preferences and Parent-Perceived AYA Communication Preferences by AYA Age at Survey

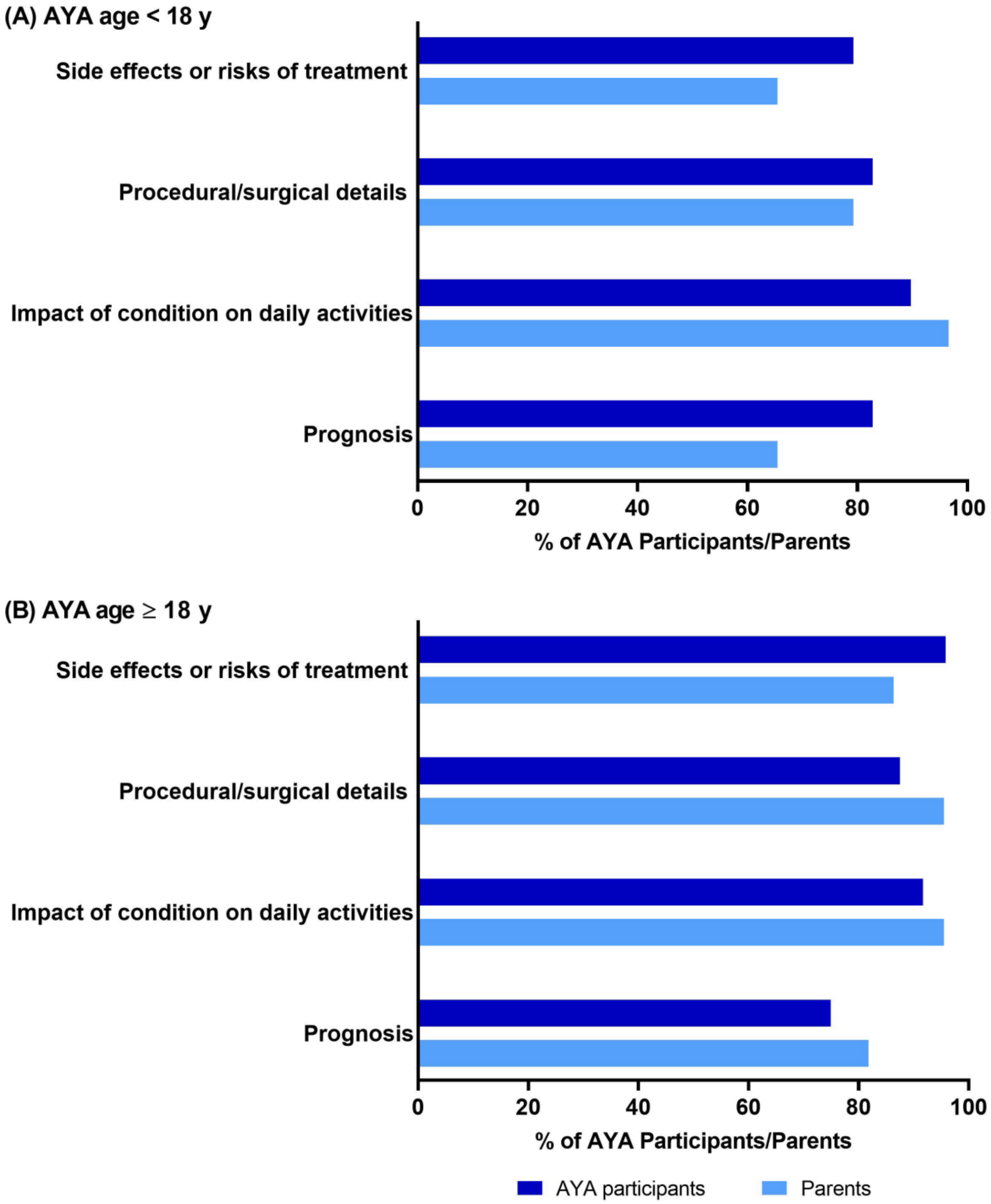

© 2023 Cousino MK et al. *JAMA Network Open*.

eFigure 2. Preferences for Involvement in End-of-Life Decision Making by AYA Age at Survey

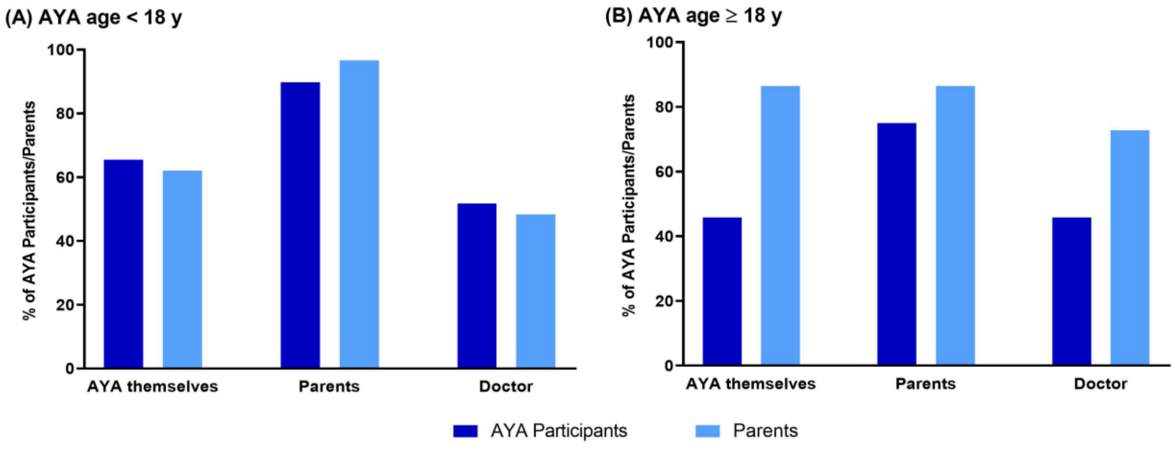

© 2023 Cousino MK et al. *JAMA Network Open*.

eFigure 3. Preferred Timing for End-of-Life Decision Making Discussions by AYA Age at Survey

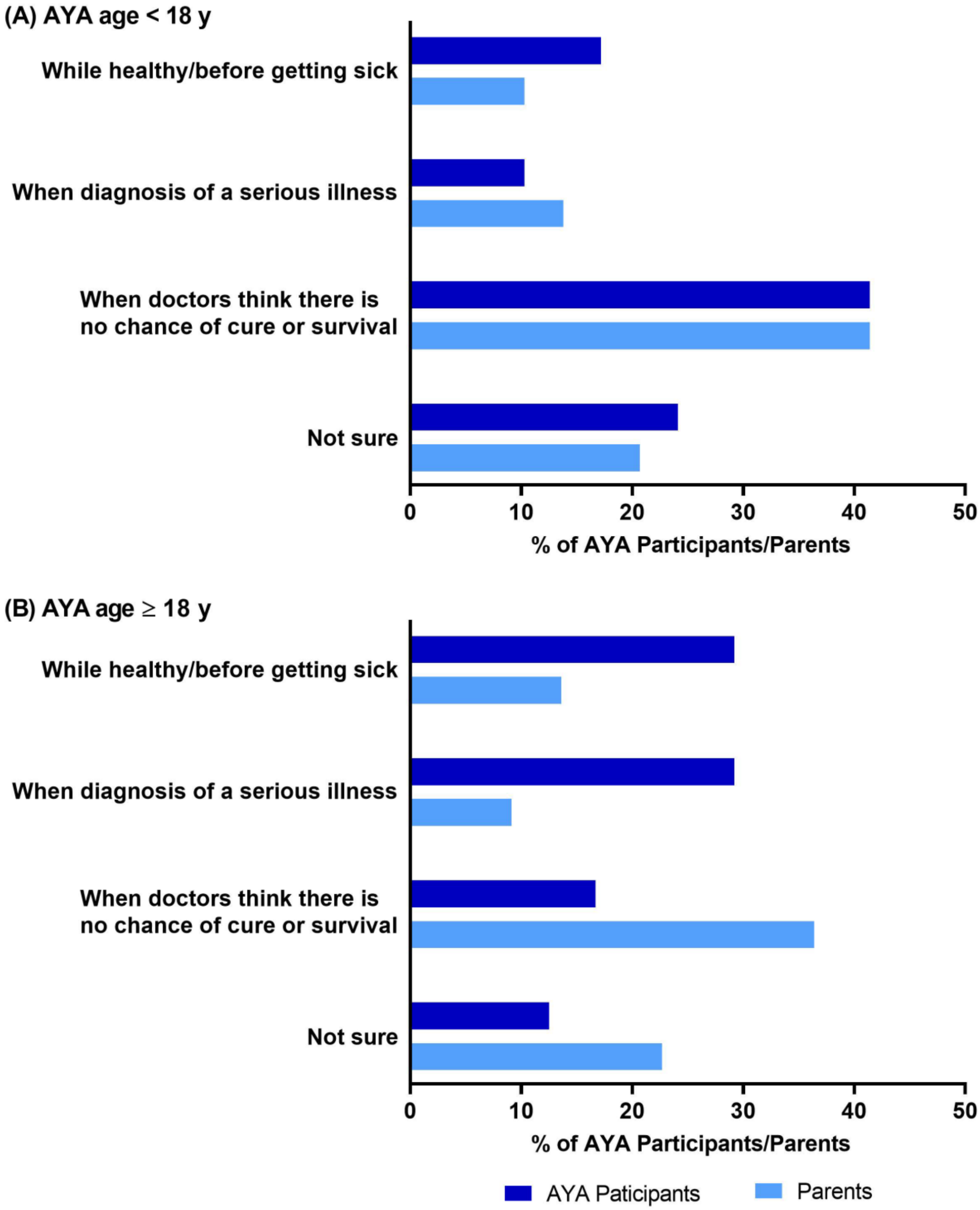

© 2023 Cousino MK et al. *JAMA Network Open*.
