# Supplementary material for: Medical and End-of-Life Decision-Making Preferences in Adolescents and Young Adults with Advanced Heart Disease and Their Parents
Source: JAMA Netw Open. 2023 May 5;6(5):e2311957. doi: 10.1001/jamanetworkopen.2023.11957 (PMC10163392; doi:10.1001/jamanetworkopen.2023.11957)
Supplement: Supplement 2. — Data Sharing Statement [file jamanetwopen-e2311957-s002.pdf]

Data Sharing Statement

Cousino. Medical and End-of-Life Decision-Making Preferences in Adolescents and Young Adults with Advanced Heart Disease and their Parents. *JAMA Netw Open*. Published May 05, 2023. doi:10.1001/jamanetworkopen.2023.11957

Data
Data available: No

Additional Information
Explanation for why data not available: Data sharing available on an individually requested basis.
